# Altered Maternal Fatty Acid Signature and Placental Transfer in Gestational Diabetes Mellitus: The Role of Fatty Acid Indices

**DOI:** 10.3390/nu18101624

**Published:** 2026-05-20

**Authors:** Magdalena Broś-Konopielko, Agnieszka Białek, Ewa Romejko-Wolniewicz, Aneta Malinowska-Polubiec, Małgorzata Białek, Krzysztof Czajkowski

**Affiliations:** 1 II Faculty and Clinic of Obstetrics and Gynaecology, Medical University of Warsaw, Karowa 2, 00-315 Warsaw, Poland; magdalena.bros-konopielko@wum.edu.pl (M.B.-K.); ewa.romejko-wolniewicz@wum.edu.pl (E.R.-W.); aneta.malinowska-polubiec@wum.edu.pl (A.M.-P.); krzysztof.czajkowski@wum.edu.pl (K.C.); 2 School of Health and Medical Sciences, VIZJA University, Okopowa 59, 01-043 Warsaw, Poland; 3 The Kielanowski Institute of Animal Physiology and Nutrition, Polish Academy of Sciences, Instytucka 3, 05-110 Jabłonna, Poland; m.bialek@ifzz.pl

**Keywords:** pregnancy, gestational diabetes mellitus, fatty acids, dietary recommendations

## Abstract

**Background:** Gestational diabetes mellitus (GDM) is associated with metabolic disturbances extending beyond glucose homeostasis, including alterations in lipid metabolism. However, evidence on the fatty acid composition of maternal serum lipids in GDM remains inconsistent, and data on placental fatty acid transfer are limited. This study aimed to explore associations between maternal serum lipid fatty acid composition, selected FA indices, and transplacental transfer of fatty acids derived from maternal serum lipids in pregnancies complicated by GDM. **Methods:** A cross-sectional study was conducted among 139 pregnant women, including 104 healthy controls and 35 women with GDM. Maternal serum and umbilical cord blood samples were collected at delivery. FA composition was analyzed using gas chromatography. Selected FA indices and the transplacental transport index (TTI) were calculated. Statistical analyses included group comparisons, multivariable models adjusted for maternal age and pre-pregnancy BMI, and false discovery rate correction. **Results:** Modest differences were observed in selected fatty acids and FA indices, particularly palmitoleic acid (C16:1) and the C16:1/C16:0 ratio. Principal component analysis suggested partial separation between groups, although substantial overlap was present. A difference in transplacental transport was observed for α-linolenic acid; however, high variability was noted. No consistent associations between reported dietary patterns and fatty acid composition of circulating serum lipids were identified. **Conclusions:** This exploratory study suggests potential differences in fatty acid composition and selected indices in GDM; however, findings should be interpreted with caution. The observed patterns may reflect late-pregnancy metabolic adaptations rather than causal mechanisms. Further studies in larger and more diverse populations are required to confirm these findings.

## 1. Introduction

Maternal nutrition during pregnancy plays a central role in supporting fetal growth and development, as well as in maintaining maternal metabolic homeostasis. Pregnancy is characterized by profound physiological adaptations, including dynamic changes in lipid metabolism, which are essential to meet the increasing energy and structural demands of the developing fetus. In early pregnancy, maternal metabolism is predominantly anabolic, favoring lipogenesis and fat storage, whereas later stages are characterized by enhanced lipolysis and increased availability of circulating lipid-derived fatty acids (FA), driven by progressive insulin resistance and hormonal regulation [1,2,3].

Fatty acids are of particular importance during pregnancy, as they serve not only as an energy source but also as key structural and functional components of cell membranes and signaling molecules. Essential fatty acids and long-chain polyunsaturated fatty acids (LC-PUFA), especially those of the *n*-3 series such as docosahexaenoic acid (DHA), are critical for fetal brain and visual development [4]. The fatty acid composition of maternal serum lipids reflects a complex interplay between dietary intake, endogenous metabolism, and placental transport, making it a biologically relevant marker of metabolic status during pregnancy.

Gestational diabetes mellitus (GDM) is one of the most common metabolic complications of pregnancy and is characterized by glucose intolerance and insulin resistance with onset or first recognition during gestation. In addition to disturbances in carbohydrate metabolism, GDM is associated with alterations in lipid metabolism, including changes in fatty acid composition of circulating serum lipids, increased lipolysis, and modified placental transfer of fatty acids derived from maternal serum lipids [5,6]. These metabolic disturbances may influence both maternal health and fetal development, as well as placental function.

Previous studies have reported differences in the fatty acid composition of maternal serum lipids between women with GDM and healthy pregnant women; however, findings remain inconsistent. Some reports suggest increased levels of saturated fatty acids (SFA) and altered proportions of polyunsaturated fatty acids (PUFA), whereas others have found no clear or consistent patterns [5,6]. These discrepancies may reflect differences in study design, timing of sampling, dietary assessment methods, and the complex regulation of lipid metabolism during pregnancy.

Importantly, most existing studies have focused on individual fatty acids or total FA classes, while relatively few have examined fatty acid indices that may better reflect metabolic processes, such as desaturase activity or endogenous lipid synthesis. Ratios such as palmitoleic to palmitic acid (C16:1/C16:0) have been proposed as markers of de novo lipogenesis and metabolic regulation, and may provide additional insight into metabolic alterations associated with insulin resistance and GDM [5].

Another critical but less explored aspect of fatty acid metabolism in pregnancy is placental transport. The placenta actively regulates the transfer of fatty acids from mother to fetus through a complex system of transport proteins, enzymes, and binding mechanisms [1,2]. This process is not purely passive and may be altered in metabolic disorders. However, data on transplacental transfer of fatty acids within maternal-fetal lipid pools in GDM remain limited, particularly in studies combining maternal and cord blood measurements.

In addition, although dietary intake is an important determinant of fatty acid availability, the relationship between reported dietary fat sources and circulating fatty acid profiles is not straightforward. The fatty acid composition of serum lipids reflects not only dietary intake but also endogenous metabolic adaptations, which may be particularly pronounced in conditions such as GDM [6]. Therefore, integrating biochemical and dietary data may help distinguish between dietary and metabolic influences on fatty acid profiles.

Given these considerations, a focused analysis of fatty acid composition of maternal serum lipids, fatty acid indices, and placental transport in GDM may provide a more comprehensive understanding of lipid metabolism in this condition.

Therefore, the aim of the present study was to compare fatty acid composition of maternal serum lipids between healthy pregnancies and those complicated by GDM, to evaluate selected fatty acid indices as potential markers of metabolic alterations, and to assess transplacental fatty acid transport using cord-to-maternal ratios. Additionally, the study explored the relationship between reported dietary fat sources and fatty acid composition of circulating serum lipids in an exploratory manner. Given the observational and exploratory nature of the study, the analysis was intended to identify associations and generate hypotheses rather than establish causal relationships.

## 2. Materials and Methods

### 2.1. Study Design and Participants

This study was designed as a cross-sectional analysis conducted among pregnant women receiving routine antenatal care at a tertiary referral centre. Participants were recruited from patients delivering at the II Faculty and Clinic of Obstetrics and Gynaecology, Medical University of Warsaw.

The study protocol was approved by the Bioethics Committee of the Medical University of Warsaw (KB 158/2010) and conducted in accordance with the Declaration of Helsinki. All participants provided written informed consent.

A total of 158 pregnant women were initially enrolled. For the purposes of the present analysis, women were classified into two groups: (i) healthy pregnancies (control group), (ii) pregnancies complicated by gestational diabetes mellitus (GDM). Women with gestational hypertension (GH) or intrahepatic cholestasis of pregnancy (ICP), as well as those with coexisting pregnancy-related conditions, were excluded from the main analysis due to limited subgroup sizes and insufficient statistical power for inferential comparisons. GDM was diagnosed according to the International Association of Diabetes and Pregnancy Study Groups (IADPSG) criteria based on a 75 g oral glucose tolerance test. Healthy participants had no diagnosed pregnancy-related metabolic or hypertensive disorders. Exclusion criteria included pre-existing diabetes mellitus, chronic hypertension, liver disease unrelated to pregnancy, multiple pregnancy, and the use of medications known to affect lipid metabolism.

Maternal anthropometric data, including height and body weight before pregnancy and at delivery, were collected, and pre-pregnancy body mass index (BMI) was calculated as weight (kg) divided by height squared (m^2^).

**Figure 1:**
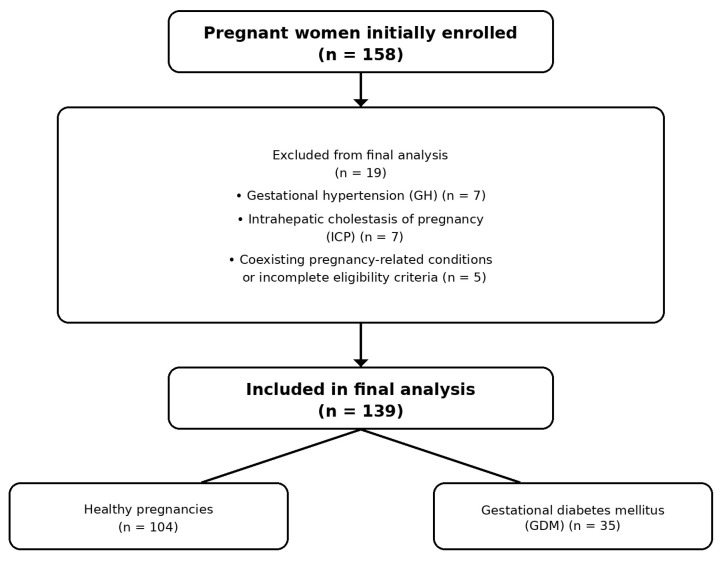
Flow diagram of participant recruitment, exclusion, and final group allocation.

A flow diagram of participant recruitment and exclusion is presented in Figure 1. Detailed inclusion and exclusion criteria are provided in Supplementary Table S1.

### 2.2. Sample Collection and Analysis of Fatty Acid Composition in Serum Lipids

Maternal venous blood samples and umbilical cord blood samples were collected at delivery. Blood samples were collected into clot tubes, centrifuged to obtain serum, and stored at −80 °C until analysis. Serum samples were thawed only once before analysis.

The fatty acid composition of total serum lipids was determined after transmethylation to fatty acid methyl esters (FAMEs), followed by gas chromatography with flame-ionization detection (GC-FID). This approach reflects fatty acids derived from the total serum lipid pool, including triglycerides, phospholipids, cholesterol esters, and a minor contribution from non-esterified fatty acids, rather than the direct measurement of circulating free fatty acids.

Three parallel serum aliquots of 100 μL were trans-esterified to prepare FAMEs according to the procedure of Bondia-Pons et al. [7], with minor modifications, as previously described [8]. No separation of individual lipid classes was performed prior to transmethylation; therefore, the obtained results represent the fatty acid composition of total serum lipids.

FAMEs analysis was performed using a GC-17A gas chromatograph (Shimadzu, Kyoto, Japan) equipped with a BPX 70 capillary column (30 m × 0.25 mm i.d., film thickness 0.20 μm; SGE, Ringwood, Australia) and a flame-ionization detector. Helium was used as the carrier gas. The injector temperature was 250 °C, and the detector temperature was 270 °C. The initial oven temperature was 140 °C for 5 min and was then increased by 4 °C/min to 240 °C.

Identification and quantification of FAMEs were performed using commercially available standards: Supelco 37 Component FAME Mix, methyl linoleate, methyl linolenate, methyl γ-linolenate, methyl arachidonate, methyl 5,8,11,14,17-eicosapentaenoate, and methyl 4,7,10,13,16,19-docosahexaenoate (Sigma-Aldrich, St. Louis, MO, USA). Individual FAMEs were identified by comparison of retention times with standards.

Individual fatty acids derived from serum lipids were expressed both as: (i) relative fatty acid composition was expressed as weight percentage (% *w*/*w*) of total identified fatty acid methyl esters (FAMEs), and (ii) absolute concentration (µg/mL). The concentration of each individual fatty acid in serum was quantified based on standard curves (µg/mL), as described previously [9].

Primary analyses were performed on maternal serum samples. Umbilical cord serum lipid-derived fatty acid concentrations were used for the assessment of transplacental transport.

### 2.3. Dietary Assessment

Dietary intake during pregnancy was assessed using a self-administered food frequency questionnaire (FFQ) designed to capture habitual intake of fat-containing food products. The questionnaire included items related to the frequency of consumption of selected food groups, including vegetable oils, animal fats, fish, dairy products, nuts, seeds, and processed foods. Responses were recorded using categorical frequency scales. The FFQ was not validated for quantitative nutrient intake and did not allow estimation of total energy or macronutrient consumption. Therefore, dietary data were analyzed qualitatively and used to describe general dietary patterns rather than precise fatty acid intake.

### 2.4. Fatty Acid Indices

To better reflect metabolic processes, selected indices derived from serum lipid fatty acid composition were calculated, including:

- C16:1/C16:0 (palmitoleic to palmitic acid ratio)
- C18:1/C18:0 ratio
- SFA/PUFA ratio
- *n*-6/*n*-3 PUFA ratio

These indices were selected as surrogate markers of endogenous lipid metabolism, including desaturase activity and the balance between saturated and unsaturated fatty acids.

### 2.5. Transplacental Transport Index (TTI)

The transplacental transport index (TTI) was calculated for selected fatty acids as the ratio of their concentration in umbilical cord serum to maternal serum:

$$TTI=\frac{FA_{cord}}{FA_{maternal}}$$

TTI was calculated for selected *n*-3 and *n*-6 fatty acids, including linoleic acid (LA), γ-linolenic acid (GLA), α-linolenic acid (ALA), arachidonic acid (AA), eicosapentaenoic acid (EPA), and docosahexaenoic acid (DHA), to assess the efficiency of placental fatty acid transfer. This index was used as an exploratory measure of relative maternal-fetal fatty acid distribution.

### 2.6. Statistical Analysis

Statistical analyses were performed using Statistica 13.3 statistical software (TIBCO Software Inc., Palo Alto, CA, USA).

Continuous variables were tested for normality using the Shapiro–Wilk test and are presented as mean ± standard deviation (SD). Categorical variables are presented as counts and percentages.

#### 2.6.1. Group Comparisons

Differences between healthy pregnancies and GDM were assessed using: (i) Student’s *t*-test for normally distributed variables or (ii) Mann–Whitney U test for non-normally distributed variables.

#### 2.6.2. Multiple Testing Correction

To account for multiple comparisons in FA analyses, false discovery rate (FDR) correction was applied using the Benjamini–Hochberg method. Adjusted *p*-values (q-values) < 0.05 were considered statistically significant.

#### 2.6.3. Multivariable Linear Regression

To assess the association between GDM and FA composition, multivariable linear regression models were constructed for selected FA and indices.

All models included:

- GDM status (binary variable)
- maternal age
- pre-pregnancy BMI.

These models were used to determine whether observed differences were independent of potential confounding factors.

#### 2.6.4. Logistic Regression

Logistic regression analysis was performed to evaluate whether selected FA indices were independently associated with the presence of GDM. Models included FA indices together with maternal age and pre-pregnancy BMI. Results are presented as regression coefficients with corresponding *p*-values and 95% confidence intervals.

#### 2.6.5. Principal Component Analysis

Principal component analysis (PCA) was performed on maternal serum lipid fatty acid composition data to identify global patterns and reduce dimensionality. Group differences in principal components were assessed using appropriate statistical tests.

#### 2.6.6. Transplacental Transport Analysis

Differences in TTI between groups were assessed using independent-sample tests. TTI values were interpreted as indicators of relative efficiency of placental fatty acid transfer.

#### 2.6.7. Significance Threshold

A *p*-value < 0.05 was considered statistically significant. For analyses involving multiple comparisons, FDR-adjusted *p*-values were used.

## 3. Results

### 3.1. Characteristics of the Study Population

The final analysis included 139 pregnant women: 104 with uncomplicated pregnancies and 35 diagnosed with gestational diabetes mellitus (GDM) (Table 1). Women with GDM were significantly older than healthy controls (33.3 ± 4.5 vs. 30.7 ± 4.2 years, *p* < 0.01). No significant differences were observed in pre-pregnancy body mass index (BMI), which was within the normal range in the majority of participants in both groups. However, body weight at delivery was lower in the GDM group compared with healthy women. Most participants had higher education and reported no smoking or alcohol consumption during pregnancy. The majority of pregnancies reached term (≥37 weeks), and neonatal outcomes, including Apgar scores, were generally favorable in both groups. These findings indicate that the study groups were largely comparable, with maternal age representing the main distinguishing baseline characteristic.

### 3.2. Fatty Acid Composition of Maternal Serum Lipids

The overall maternal serum fatty acid profile is presented in Table 2. Analysis of the fatty acid composition of maternal serum lipids revealed differences between healthy pregnancies and those complicated by GDM.

Women with GDM exhibited significantly lower proportions of selected saturated and monounsaturated fatty acids, including myristic acid (C14:0), palmitic acid (C16:0), and palmitoleic acid (C16:1), as well as lower total saturated fatty acids (SFA). Among these, palmitoleic acid (C16:1) showed the most pronounced difference between groups.

After correction for multiple comparisons, the most robust differences were observed for C16:1 and total SFA. Other lipid-derived fatty acids showed nominal differences but did not remain significant after adjustment.

In contrast, no consistent or statistically robust differences were observed in total polyunsaturated fatty acids (PUFA), *n*-3 PUFA, *n*-6 PUFA, or their ratios after correction for multiple testing.

### 3.3. Adjusted Analysis of Fatty Acids

To account for potential confounding factors, linear regression models were constructed including maternal age and pre-pregnancy BMI. After adjustment, several fatty acids remained significantly associated with GDM, including C16:1, C14:0, C14:1, C17:1, and total SFA. Detailed results of the adjusted linear regression models are presented in Table 3. Among these, C16:1 remained the most consistently associated fatty acid. In contrast, differences observed in some fatty acids in unadjusted analyses (e.g., C16:0 or selected PUFA) were attenuated after adjustment, suggesting that they may be partly explained by baseline characteristics. These findings indicate that specific alterations in fatty acid composition persist independently of maternal age and BMI.

### 3.4. Fatty Acid Indices

To better capture metabolic patterns, selected fatty acid ratios were analyzed. The ratio of palmitoleic to palmitic acid (C16:1/C16:0) was significantly lower in women with GDM and remained strongly associated with GDM after adjustment for maternal age and BMI. Similarly, the SFA/PUFA ratio was lower in GDM, although its association was weaker after adjustment. The ratio C18:1/C18:0 was higher in GDM, suggesting altered desaturation processes. Among these indices, C16:1/C16:0 showed the strongest association with GDM among the analyzed indices differentiating GDM from healthy pregnancies. Results of the adjusted analysis for selected fatty acid indices are presented in Table 4.

### 3.5. Principal Component Analysis

**Figure 2:**
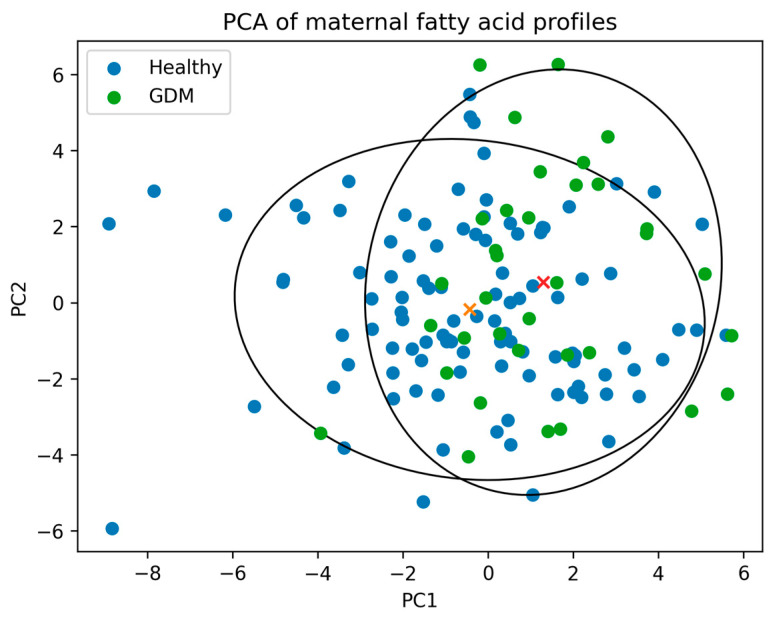
Principal component analysis (PCA) of fatty acid composition of maternal serum lipids. Scatter plot of the first two principal components (PC1 and PC2) showing partial separation between pregnancies complicated by gestational diabetes mellitus (GDM) and healthy controls. Ellipses represent the distribution of each group, and crosses indicate group centroids.

Principal component analysis (PCA) was performed to explore overall patterns in fatty acid composition. The first principal component (PC1), explaining approximately 20% of total variance, differed significantly between groups (*p* < 0.001). The distribution of study groups in the PCA space is shown in Figure 2. Fatty acids contributing most strongly to PC1 included C16:1, total SFA, and selected *n*-6 PUFA. Detailed PCA loadings are presented in Supplementary Table S9. No significant group differences were observed for the second principal component. These findings suggest a partial separation of fatty acid composition patterns between groups, although the substantial overlap indicates that PCA results should be interpreted as exploratory.

### 3.6. Dietary Assessment

Dietary data were analyzed qualitatively based on food frequency questionnaire responses. Detailed dietary data are presented in Supplementary Tables S2–S8. No consistent or statistically significant associations between reported dietary fat sources and fatty acid composition patterns were observed after correction for multiple comparisons. These findings indicate that fatty acid composition of circulating serum lipids in late pregnancy may be influenced more strongly by endogenous metabolic processes than by reported dietary intake.

### 3.7. Absolute Concentrations of Selected Lipid-Derived Fatty Acids

No significant differences were observed between groups in absolute concentrations of key *n*-3 and *n*-6 fatty acids in maternal serum after correction for multiple comparisons. This suggests that relative fatty acid composition, rather than absolute concentrations, may better reflect metabolic alterations associated with GDM.

### 3.8. Logistic Regression Analysis

Logistic regression analysis was performed to assess whether selected fatty acid indices were associated with GDM after adjustment for maternal age and BMI. The C16:1/C16:0 ratio was significantly associated with GDM after adjustment for maternal age and BMI (*p* = 0.0019). Results of the logistic regression analysis are summarized in Table 5. Lower values of this ratio were associated with higher odds of GDM. In contrast, the SFA/PUFA ratio, maternal age, and BMI were not independently associated with GDM in this model.

### 3.9. Transplacental Fatty Acid Transport

The transplacental transport index (TTI) was calculated for selected fatty acids. No significant differences were observed for linoleic acid (LA) or γ-linolenic acid (GLA). However, TTI for α-linolenic acid (ALA) differed in adjusted analyses, although this association did not remain significant after FDR correction. Transplacental transport indices for selected fatty acids are presented in Table 6. This may suggest differences in placental transfer of selected fatty acids in GDM; however, the large variability observed for ALA indicates that this result should be interpreted with caution.

## 4. Discussion

### 4.1. Principal Findings

The present study provides an exploratory analysis of fatty acid composition of maternal serum lipids, selected FA indices, and transplacental transfer of fatty acids derived from maternal serum lipids in pregnancies complicated by GDM. The main findings indicate modest differences in selected FA and FA indices between women with GDM and healthy controls, particularly for palmitoleic acid (C16:1) and the C16:1/C16:0 ratio. In addition, the analysis suggested a difference in the TTI for α-linolenic acid (ALA). However, these findings should be interpreted with caution due to the cross-sectional design, the limited number of GDM cases, and the exploratory nature of the study.

### 4.2. Alterations in Maternal Serum Lipid Fatty Acid Composition in GDM

Pregnancy is associated with progressive changes in lipid metabolism, including increased lipolysis and increased circulating lipid-derived fatty acids in late gestation, which serve to support fetal growth [1,2,3]. In GDM, these physiological processes may be further modified by insulin resistance and altered hormonal signaling, leading to dysregulation of lipid metabolism [5,6]. Additionally, hormonal regulation plays a central role in shaping metabolic adaptations during pregnancy. In particular, human placental lactogen (hPL), a key placental hormone, contributes to the progressive development of maternal insulin resistance, ensuring an adequate supply of nutrients to the fetus. hPL promotes lipolysis and increases circulating non-esterified fatty acids (NEFA), while simultaneously modulating maternal glucose metabolism by reducing peripheral glucose utilization [1,3]. In the context of GDM, alterations in hPL signaling or its metabolic effects may contribute to lipid mobilization and may partly explain differences in FA profiles observed in late pregnancy; however, this mechanism was not directly assessed in the present study [10,11].

In the present study, women with GDM exhibited lower proportions of selected SFA and MUFA, particularly C16:1. Previous studies have reported both increases and decreases in specific fatty acids in GDM, and the overall picture remains inconsistent [5,6,12]. These discrepancies may be related to differences in study populations, timing of sampling, analytical methods, dietary assessment, and the complex regulation of lipid metabolism during pregnancy.

Our findings suggest that relative fatty acid composition may provide information on metabolic status in GDM, but this interpretation should remain cautious. We did not observe significant differences in absolute concentrations of major *n*-3 and *n*-6 fatty acids after correction for multiple comparisons, which may indicate that compositional measures and FA indices are more sensitive to subtle metabolic differences than absolute concentrations. However, absolute FA concentrations may also be affected by pregnancy-related plasma volume expansion, which was not directly accounted for in the present analysis.

Recent studies have emphasized the importance of lipidomic profiling in GDM, highlighting that subtle shifts in fatty acid composition may reflect underlying metabolic disturbances, including altered insulin signaling and hepatic lipid metabolism [13,14]. Our results are broadly consistent with this perspective, although they should be considered preliminary and require confirmation in larger cohorts.

### 4.3. Fatty Acid Indices as Markers of Metabolic Dysregulation

A key finding of this study is the association between the C16:1/C16:0 ratio and GDM. This ratio is commonly interpreted as an indirect surrogate marker of stearoyl-CoA desaturase-1 (SCD1) activity and de novo lipogenesis. However, because SCD1 activity, gene expression, or related enzymatic pathways were not directly measured in this study, this interpretation should be considered hypothetical rather than mechanistically proven.

Palmitoleic acid (C16:1) has been proposed as a lipokine involved in metabolic regulation, with potential roles in insulin sensitivity and lipid homeostasis. Alterations in its relative abundance may reflect impaired desaturation processes or altered hepatic lipid metabolism, both of which are implicated in insulin resistance [13,15]. Nevertheless, the magnitude of the observed differences was modest, and their biological or clinical relevance remains uncertain.

The observed reduction in the C16:1/C16:0 ratio in GDM may suggest altered regulation of lipid synthesis or desaturation pathways. Importantly, this association remained significant after adjustment for maternal age and BMI, but residual confounding cannot be excluded. Therefore, this ratio should not be interpreted as a validated biomarker, but rather as a parameter that may warrant further investigation in larger and preferably longitudinal studies.

These findings support the concept that FA indices may provide complementary information to individual FA values, particularly when exploring functional aspects of lipid metabolism. Similar approaches have been increasingly used in nutritional and metabolic research to capture broader metabolic patterns [13,16].

### 4.4. Placental Fatty Acid Transport in GDM

An important observation of this study was the difference in TTI for ALA in pregnancies complicated by GDM. While this finding is of potential interest, it should be interpreted cautiously. The relatively large standard deviation observed for ALA TTI in the GDM group suggests substantial variability and raises the possibility of outliers, measurement variability, or a chance finding.

The placenta plays a central role in regulating fetal nutrient supply, including fatty acids, through a complex network of transport proteins, binding proteins, and enzymatic systems [1,2]. Fatty acid transfer is a regulated process influenced by both maternal metabolic status and placental function. However, the present study did not include direct measurements of placental transporters, such as FATP or MFSD2a, nor enzymes involved in lipid metabolism. Therefore, the observed TTI differences cannot be attributed to specific placental mechanisms.

Our findings may suggest that GDM is associated with differences in the relative transfer of selected fatty acids, but they do not establish differences in relative maternal–fetal fatty acid distribution as a causal mechanism. Interestingly, no comparable differences were observed for LA or GLA, suggesting that the ALA finding may be selective. However, because this was an isolated result and the number of comparisons was high, it requires confirmation.

Recent studies have suggested that GDM may be associated with changes in placental lipid transport proteins and fatty acid metabolism, which may influence fetal exposure to lipids [5,6]. Our results are generally in line with these observations, but should be considered preliminary. Similar alterations in placental lipid transport and cord blood fatty acid composition in GDM have also been reported previously [17,18].

### 4.5. Diet Versus Endogenous Metabolism

Despite the inclusion of dietary data, we did not observe consistent associations between reported dietary fat intake and circulating fatty acid profiles after correction for multiple comparisons. However, this finding should not be interpreted as evidence that diet does not influence FA status. Dietary intake was assessed using a qualitative, non-validated food frequency questionnaire, which did not allow quantitative estimation of nutrient intake, total energy intake, or macronutrient composition.

Therefore, the present data do not allow a clear distinction between the relative contribution of dietary intake and endogenous metabolism to fatty acid composition of circulating serum lipids. While diet is an important source of fatty acids, circulating FA composition reflects the combined effects of dietary intake, endogenous synthesis, mobilization of maternal fat stores, hormonal regulation, and placental transfer.

In conditions such as GDM, metabolic alterations may influence serum lipid fatty acid composition, but the extent to which they override dietary factors cannot be determined from the present study. Similar observations have been reported in recent studies, where metabolic state rather than diet alone was identified as an important determinant of serum lipid fatty acid composition in pregnancy [14,16]. Intervention studies evaluating omega-3 supplementation in women with GDM further support the relevance of altered fatty acid metabolism in this condition [19], while recent work in pregnant populations has highlighted the importance of omega-3 status more broadly [20].

### 4.6. Clinical and Biological Implications

The identification of selected FA patterns and indices associated with GDM may have biological relevance, although this remains to be confirmed and any clinical implications should be considered preliminary. In particular, the C16:1/C16:0 ratio should not be regarded as a biomarker at this stage. Rather, it may represent a candidate parameter for further investigation in larger, independent cohorts with longitudinal sampling and validation analyses.

Similarly, the observed difference in ALA TTI may suggest that maternal–fetal FA transfer deserves further investigation in GDM. However, without direct measurement of placental transporters, enzyme activity, or longitudinal maternal and fetal FA trajectories, these findings remain hypothesis-generating.

The integration of FA profiling with indices and transport measures may help characterize lipid metabolism during pregnancy. Future studies should include larger and more diverse populations, quantitative dietary assessment, information on gestational weight gain, physical activity, insulin use or other antidiabetic treatment, and neonatal outcomes.

### 4.7. Strengths and Limitations

The strengths of this study include the combined analysis of fatty acid composition of maternal and cord blood serum lipids, the use of FA indices to capture broader metabolic patterns, and the application of multivariable models with adjustment for maternal age and pre-pregnancy BMI. The use of FDR correction also reduced the risk of false-positive findings related to multiple comparisons.

However, several limitations should be acknowledged. First, the cross-sectional design precludes causal inference, and reverse causality or residual confounding cannot be ruled out. Second, FA measurements were performed at delivery and may not reflect earlier metabolic changes during pregnancy, including the period of GDM diagnosis or placental development. Third, although the study focused on two groups to improve interpretability, the number of GDM cases remained limited, which may affect statistical power and the stability of multivariable estimates.

Fourth, dietary assessment was based on a non-validated qualitative FFQ and did not allow quantitative estimation of nutrient or energy intake. Fifth, potentially important confounders, including gestational weight gain, physical activity, insulin use, and antidiabetic treatment, were not included in the analysis. Sixth, absolute FA concentrations may be influenced by plasma volume expansion during pregnancy, which was not directly assessed. In addition, the present analysis was based on total serum lipid fractions without separation of individual lipid classes. Since fatty acids are not equally distributed among triglycerides, phospholipids, cholesterol esters, and non-esterified fatty acids, and may also differ in placental transfer dynamics depending on lipid class, these findings should be interpreted as reflecting the overall serum lipid fatty acid profile rather than specific lipidomic compartments. Finally, the study population was relatively homogeneous and highly educated, which may limit generalizability.

### 4.8. Future Directions

Future studies should confirm these findings in larger, independent, and preferably longitudinal cohorts. Such studies should include quantitative dietary assessment, repeated FA measurements across pregnancy, direct evaluation of placental transporters and lipid-related enzymes, and analyses linking maternal and cord blood FA profiles with neonatal outcomes. This would allow a more precise assessment of whether the observed FA patterns are clinically relevant and whether they reflect causal mechanisms, adaptive responses, or late-pregnancy metabolic consequences of GDM. Overall, the present findings should be considered exploratory and hypothesis-generating.

## 5. Conclusions

In this exploratory study, modest differences in fatty acid composition of maternal serum lipids and selected fatty acid indices were observed between pregnancies complicated by gestational diabetes mellitus (GDM) and healthy controls. In particular, alterations in C16:1 and the C16:1/C16:0 ratio, as well as a difference in the transplacental transport index for α-linolenic acid, were identified.

However, due to the cross-sectional design, relatively limited sample size, and methodological constraints, these findings should be interpreted with caution. No causal relationships can be established, and the biological and clinical relevance of the observed differences remains uncertain.

Overall, the results should be considered exploratory and hypothesis-generating. Further studies, particularly those with longitudinal design, larger and more diverse populations, and comprehensive dietary and metabolic assessment, are needed to confirm these observations and to better understand their underlying mechanisms.

## Figures and Tables

**Table 1:** Characteristics of the study population.

Characteristic	Healthy (*n* = 104)	GDM (*n* = 35)	*p*
**Continuous characteristics**			
Age, years	30.72 ± 4.23	33.31 ± 4.49	0.0041
Height, cm	167.32 ± 5.91	165.71 ± 6.67	0.2127
Pre-pregnancy weight, kg	63.14 ± 11.46	62.51 ± 13.01	0.8018
Weight at delivery, kg	78.72 ± 12.85	72.14 ± 12.29	0.0091
Pre-pregnancy BMI, kg/m^2^	22.53 ± 3.74	22.71 ± 4.10	0.8211
Neonatal head circumference, cm	34.79 ± 1.39	34.40 ± 1.68	0.2208
Neonatal chest circumference, cm	33.82 ± 1.63	33.93 ± 1.91	0.7757
Birth weight, g	3505.90 ± 399.89	3535.57 ± 452.73	0.7314
Birth length, cm	54.67 ± 2.92	54.54 ± 2.87	0.8181
Ponderal index	21.67 ± 2.78	21.95 ± 2.60	0.5856
**Categorical characteristics**			
Higher education, *n* (%)	76 (76.0%)	27 (77.1%)	1.0000
Nulliparous, *n* (%)	48 (46.2%)	9 (25.7%)	0.0463
History of miscarriage, *n* (%)	18 (17.3%)	11 (31.4%)	0.0932
Term delivery (≥37 weeks), *n* (%)	101 (97.1%)	34 (97.1%)	1.0000
Cesarean section, *n* (%)	26 (25.0%)	12 (34.3%)	0.2838
Male infant, *n* (%)	43 (41.3%)	17 (48.6%)	0.5545
Child abnormalities, *n* (%)	16 (15.4%)	8 (22.9%)	0.3119
Smoking during pregnancy, *n* (%)	1 (1.0%)	0 (0.0%)	1.0000
Alcohol during pregnancy, *n* (%)	1 (1.0%)	0 (0.0%)	1.0000

Continuous data are mean ± SD. Categorical data are *n* (%). *p*-values are from Welch *t*-test (continuous) or Fisher exact test (categorical).

**Table 2:** Maternal serum fatty acid composition (%) in healthy pregnancies and GDM.

Fatty Acid	Healthy Mean	Healthy SD	GDM Mean	GDM SD	*p* Raw	FDR	Cohen d
C12:0	0.095	0.042	0.085	0.051	0.2774	0.4895	−0.236
C14:0	1.00	0.29	0.83	0.29	0.0031	0.0157	−0.605
C15:0	0.215	0.053	0.209	0.051	0.5505	0.7614	−0.115
C16:0	23.2	2.2	22.2	1.4	0.0029	0.0157	−0.479
C17:0	0.200	0.038	0.21	0.05	0.274	0.4895	0.247
C18:0	4.23	0.50	4.01	0.56	0.045	0.1351	−0.423
C20:0	0.036	0.043	0.036	0.048	0.922	0.9353	−0.02
C22:0	0.092	0.036	0.078	0.022	0.0089	0.0383	−0.408
C23:0	0.063	0.036	0.059	0.031	0.4848	0.7614	−0.127
C24:0	0.124	0.04	0.121	0.052	0.7196	0.8304	−0.08
Total SFA	29.3	2.5	27.9	1.5	0.0001	0.0018	−0.612
C14:1	0.042	0.016	0.033	0.013	0.0023	0.0157	−0.559
C16:1	2.30	0.73	1.74	0.49	0	0.0001	−0.823
C17:1	0.166	0.045	0.144	0.029	0.0012	0.0125	−0.524
Oleic acid (C18:1n9)	20.1	2.3	21.1	3.1	0.095	0.2192	0.383
C20:1	0.023	0.013	0.024	0.013	0.6839	0.8206	0.079
C22:1	0.022	0.019	0.022	0.014	0.8342	0.8938	−0.036
Total MUFA	22.7	2.5	23.1	3.1	0.5086	0.7614	0.147
*trans*-LA isomer (t9,t12 C18:2)	0.075	0.026	0.064	0.023	0.0227	0.0852	−0.426
LA (C18:2n6)	18.9	2.6	19.9	2.5	0.0523	0.137	0.374
GLA (C18:3n6)	0.177	0.066	0.167	0.089	0.5401	0.7614	−0.14
ALA (C18:3n3)	0.61	0.18	0.60	0.24	0.7819	0.8687	−0.063
C20:2	0.171	0.044	0.166	0.043	0.5837	0.7614	−0.106
AA (C20:4n6)	3.86	0.80	4.05	0.75	0.2004	0.4294	0.245
EPA (C20:5n3)	0.30	0.24	0.33	0.33	0.5827	0.7614	0.125
DHA (C22:6n3)	1.72	0.42	1.69	0.36	0.6691	0.8206	−0.077
Total PUFA	25.8	2.7	27.0	3.0	0.0548	0.137	0.406
Total *n*-3 PUFA	2.63	0.66	2.62	0.68	0.9353	0.9353	−0.016
Total *n*-6 PUFA	23.2	2.7	24.3	2.8	0.0388	0.1294	0.421
*n*-6/*n*-3 PUFA ratio	9.3	2.4	9.8	2.4	0.2595	0.4895	0.224

Negative Cohen d values indicate lower values in GDM. False discovery rate (FDR) was controlled with the Benjamini–Hochberg procedure.

**Table 3:** Multivariable linear regression for selected fatty acids and indices.

Variable	Adjusted β for GDM	95% CI Low	95% CI High	*p* Raw	FDR	Adjusted R^2^
C16:1/C16:0	−0.02	−0.03	−0.01	0.0001	0.0012	0.12
C16:1	−0.517	−0.786	−0.248	0.0002	0.0012	0.116
C14:0	−0.175	−0.289	−0.06	0.003	0.0109	0.096
C18:1/C18:0	0.634	0.206	1.061	0.004	0.0109	0.05
SFA	−1.233	−2.146	−0.321	0.0085	0.0187	0.05
C17:1	−0.022	−0.039	−0.005	0.0112	0.0194	0.048
SFA/PUFA	−0.09	−0.16	−0.02	0.0124	0.0194	0.04
C14:1	−0.007	−0.013	−0.001	0.0209	0.0288	0.099
C22:0	−0.015	−0.029	−0.001	0.0328	0.0401	0.013
C16:0	−0.783	−1.595	0.028	0.0584	0.0643	0.023
n6/n3 PUFA	0.546	−0.378	1.47	0.2445	0.2445	0.018

Each model included GDM status, maternal age, and pre-pregnancy BMI. β denotes the adjusted difference associated with GDM.

**Table 4:** Absolute concentrations of selected fatty acids derived from maternal and cord serum lipids (µg/mL).

Fatty Acid	Maternal Healthy Mean ± SD	Maternal GDM Mean ± SD	*p* Raw	FDR	Cohen d	Cord Healthy Mean ± SD	Cord GDM Mean ± SD	*p* Raw	FDR	Cohen d
LA	965.47 ± 360.87	1027.09 ± 335.53	0.3602	0.8645	0.174	83.26 ± 38.33	85.69 ± 29.25	0.6971	0.9184	0.067
GLA	9.26 ± 8.47	8.74 ± 11.32	0.8034	0.9184	−0.056	2.44 ± 1.46	2.40 ± 1.24	0.8611	0.9184	−0.032
ALA	27.66 ± 16.03	24.43 ± 14.35	0.2673	0.8645	−0.207	1.20 ± 1.68	1.42 ± 1.98	0.5631	0.9184	0.124
AA	190.60 ± 71.70	205.74 ± 69.46	0.2731	0.8645	0.213	92.70 ± 32.53	99.19 ± 33.41	0.3217	0.8645	0.198
EPA	10.50 ± 11.47	11.46 ± 11.11	0.6599	0.9184	0.085	1.84 ± 1.40	1.82 ± 1.28	0.9184	0.9184	−0.019
DHA	108.32 ± 36.37	107.47 ± 37.19	0.9071	0.9184	−0.023	40.01 ± 14.58	36.25 ± 10.34	0.0996	0.8645	−0.275

Maternal and cord serum lipid-derived fatty acid concentrations are presented side-by-side to facilitate comparison of maternal–fetal fatty acid distribution.

**Table 5:** Logistic regression model for GDM.

Predictor	Odds Ratio	95% CI Low	95% CI High	*p*
C16:1/C16:0 (per 0.01 increase)	0.661	0.503	0.869	0.0031
SFA/PUFA (per 0.1 increase)	0.976	0.677	1.408	0.8978
Age, years	1.151	1.03	1.285	0.0129
Pre-pregnancy BMI, kg/m^2^	0.967	0.868	1.076	0.5338

Model predictors: C16:1/C16:0 (per 0.01 increase), SFA/PUFA (per 0.1 increase), maternal age, and pre-pregnancy BMI.

**Table 6:** Transplacental transport index (TTI) for selected fatty acids.

TTI	Healthy Mean	Healthy SD	GDM Mean	GDM SD	*p* Raw	FDR	Adjusted β for GDM	95% CI Low	95% CI High	Adjusted *p*	Adjusted FDR
TTI LA	0.091	0.038	0.089	0.038	0.7931	0.7931	0.002	−0.014	0.017	0.8246	0.9514
TTI GLA	0.414	0.478	0.46	0.371	0.5539	0.7465	0.006	−0.177	0.188	0.9514	0.9514
TTI ALA	0.071	0.172	0.395	1.47	0.2021	0.7465	0.379	0.074	0.683	0.0152	0.091
TTI AA	0.527	0.196	0.506	0.153	0.5183	0.7465	−0.013	−0.089	0.063	0.7347	0.9514
TTI EPA	0.212	0.142	0.242	0.346	0.6221	0.7465	0.023	−0.063	0.11	0.5938	0.9514
TTI DHA	0.391	0.144	0.371	0.153	0.4973	0.7465	−0.009	−0.068	0.05	0.7645	0.9514

TTI was calculated as the cord blood to maternal serum concentration ratio. Adjusted models included maternal age and pre-pregnancy BMI. TTI values should be interpreted in conjunction with maternal and cord serum lipid-derived fatty acid concentrations presented in Table 4.

## Data Availability

The data presented in this study are available on reasonable request from the corresponding author. The data are not publicly available due to privacy and ethical restrictions related to clinical participant information.

## Supplementary Materials

The following supporting information can be downloaded at: https://www.mdpi.com/article/10.3390/nu18101624/s1, Table S1: Inclusion and exclusion criteria applied in participant selection. Table S2: Frequency of consumption of selected vegetable oils in healthy pregnancies and pregnancies complicated by gestational diabetes mellitus (GDM). Table S3: Frequency of fish consumption in healthy pregnancies and pregnancies complicated by gestational diabetes mellitus (GDM). Table S4: Frequency of consumption of nuts in healthy pregnancies and pregnancies complicated by gestational diabetes mellitus (GDM). Table S5: Frequency of consumption of fats and fat-containing products in healthy pregnancies and pregnancies complicated by gestational diabetes mellitus (GDM). Table S6: Frequency of consumption of eggs in healthy pregnancies and pregnancies complicated by gestational diabetes mellitus (GDM). Table S7: Frequency of consumption of cereals and seeds in healthy pregnancies and pregnancies complicated by gestational diabetes mellitus (GDM). Table S8: Frequency of consumption of selected food products not included in previous categories in healthy pregnancies and pregnancies complicated by gestational diabetes mellitus (GDM). Table S9: PCA loadings for fatty acid composition of maternal serum lipids.

## Abbreviations

The following abbreviations are used in this manuscript:

AA	Arachidonic acid
ALA	α-Linolenic acid
BMI	Body Mass Index
DHA	Docosahexaenoic acid
EPA	Eicosapentaenoic acid
FA	Fatty acids within serum lipid fractions
FAMEs	Fatty acid methyl esters
FFQ	Food-frequency questionnaire
GC-FID	Gas chromatography–flame ionisation detector
GDM	Gestational diabetes mellitus
GLA	γ-Linolenic acid
LA	Linoleic acid
LC-PUFA	Long-chain polyunsaturated fatty acids
MUFA	Monounsaturated fatty acids
*n*-3 PUFA	Omega-3 polyunsaturated fatty acids
*n*-6 PUFA	Omega-6 polyunsaturated fatty acids
NEFA	Non-esterified fatty acids
OL	Oleic acid
PCA	Principal Component Analysis
PUFA	Polyunsaturated fatty acids
SCD1	Stearoyl-CoA desaturase-1
SFA	Saturated fatty acids
TTI	Transplacental transport index
