# Supplementary material for: Altered Maternal Fatty Acid Signature and Placental Transfer in Gestational Diabetes Mellitus: The Role of Fatty Acid Indices
Source: Nutrients. 2026 May 20;18(10):1624. doi: 10.3390/nu18101624 (PMC13209372; doi:10.3390/nu18101624)
Supplement: Supplementary file 1 [file nutrients-18-01624-s001.zip › nutrients-4296265-supplementary.pdf]

# Supplementary Materials

**Table S1.** Inclusion and exclusion criteria applied in participant selection.

| **Inclusion criteria** | **Exclusion criteria** |
|---|---|
| Singleton pregnancy | Multiple pregnancy |
| Maternal age ≥18 years | Pre-existing diabetes mellitus |
| Availability of maternal serum sample | Chronic hypertension |
| Availability of umbilical cord blood sample | Liver disease unrelated to pregnancy |
| Availability of complete clinical data | Coexisting pregnancy complications |
| Written informed consent | Missing biological material |
| Delivery at study center | Incomplete clinical data |

**Table S2.** Frequency of consumption of selected vegetable oils in healthy pregnancies and pregnancies complicated by gestational diabetes mellitus (GDM).

| | | **Healthy pregnancies n = 104** | | **GDM n = 35** | |
|---|---|---|---|---|---|
| olive | | n | % | n | % |
| | everyday | 12 | 11.5 | 7 | 20.0 |
| | 4-6 times a week | 6 | 5.8 | 1 | 2.9 |
| | 2-3 times a week | 10 | 9.6 | 8 | 22.9 |
| | once a week | 14 | 13.5 | 2 | 5.7 |
| | once a two weeks | 13 | 12.5 | 4 | 11.4 |
| | never | 29 | 27.9 | 8 | 22.9 |
| | no data | 20 | 19.2 | 5 | 14.3 |
| sunflower | | | | | |
| | everyday | 2 | 1.9 | 3 | 8.6 |
| | 4-6 times a week | 0 | 0.0 | 0 | 0.0 |
| | 2-3 times a week | 13 | 12.5 | 2 | 5.7 |
| | once a week | 15 | 14.4 | 0 | 0.0 |
| | once a two weeks | 11 | 10.6 | 4 | 11.4 |
| | never | 43 | 41.3 | 21 | 60.0 |
| | no data | 20 | 19.2 | 5 | 14.3 |
| rapeseed | | | | | |
| | everyday | 5 | 4.8 | 5 | 14.3 |
| | 4-6 times a week | 8 | 7.7 | 0 | 0.0 |
| | 2-3 times a week | 12 | 11.5 | 3 | 8.6 |
| | once a week | 7 | 6.7 | 6 | 17.1 |
| | once a two weeks | 5 | 4.8 | 2 | 5.7 |
| | never | 47 | 45.2 | 14 | 40.0 |
| | no data | 20 | 19.2 | 5 | 14.3 |
| linseed | | | | | |
| | everyday | 1 | 1.0 | 0 | 0.0 |

| | | | | | |
|---|---|---|---|---|---|
| | 4-6 times a week | 1 | 1.0 | 2 | 5.7 |
| | 2-3 times a week | 0 | 0.0 | 1 | 2.9 |
| | once a week | 0 | 0.0 | 0 | 0.0 |
| | once a two weeks | 1 | 1.0 | 0 | 0.0 |
| | never | 81 | 77.9 | 27 | 77.1 |
| | no data | 20 | 19.2 | 5 | 14.3 |
| grapeseed | | | | | |
| | everyday | 0 | 0.0 | 1 | 2.9 |
| | 4-6 times a week | 1 | 1.0 | 0 | 0.0 |
| | 2-3 times a week | 1 | 1.0 | 0 | 0.0 |
| | once a week | 0 | 0.0 | 1 | 2.9 |
| | once a two weeks | 1 | 1.0 | 1 | 2.9 |
| | never | 81 | 77.9 | 27 | 77.1 |
| | no data | 20 | 19.2 | 5 | 14.3 |
| sesame | | | | | |
| | everyday | 0 | 0.0 | 0 | 0.0 |
| | 4-6 times a week | 0 | 0.0 | 0 | 0.0 |
| | 2-3 times a week | 0 | 0.0 | 0 | 0.0 |
| | once a week | 1 | 1.0 | 0 | 0.0 |
| | once a two weeks | 0 | 0.0 | 0 | 0.0 |
| | never | 83 | 79.8 | 30 | 85.7 |
| | no data | 20 | 19.2 | 5 | 14.3 |
| soybean | | | | | |
| | everyday | 0 | 0.0 | 0 | 0.0 |
| | 4-6 times a week | 0 | 0.0 | 0 | 0.0 |
| | 2-3 times a week | 0 | 0.0 | 0 | 0.0 |
| | once a week | 1 | 1.0 | 0 | 0.0 |
| | once a two weeks | 1 | 1.0 | 0 | 0.0 |
| | never | 81 | 77.9 | 30 | 85.7 |
| | no data | 21 | 20.2 | 5 | 14.3 |

**Table S3.** Frequency of fish consumption in healthy pregnancies and pregnancies complicated by gestational diabetes mellitus (GDM).

| | | **Healthy pregnancies** | | **GDM** | |
|---|---|---|---|---|---|
| | | **n = 104** | | **n = 35** | |
| salmon | | n | % | n | % |
| | everyday | 0 | 0.0 | 0 | 0.0 |
| | 4-6 times a week | 0 | 0.0 | 0 | 0.0 |
| | 2-3 times a week | 5 | 4.8 | 3 | 8.6 |
| | once a week | 6 | 5.8 | 4 | 11.4 |
| | once a two weeks | 27 | 26.0 | 8 | 22.9 |
| | never | 47 | 45.2 | 15 | 42.9 |
| | no data | 19 | 18.3 | 5 | 14.3 |
| tuna | | | | | |
| | everyday | 0 | 0.0 | 0 | 0.0 |
| | 4-6 times a week | 0 | 0.0 | 0 | 0.0 |
| | 2-3 times a week | 2 | 1.9 | 0 | 0.0 |

| | | | | | |
|---|---|---|---|---|---|
| | once a week | 4 | 3.8 | 2 | 5.7 |
| | once a two weeks | 8 | 7.7 | 5 | 14.3 |
| | never | 71 | 68.3 | 23 | 65.7 |
| | no data | 19 | 18.3 | 5 | 14.3 |
| mackerel | | | | | |
| | everyday | 0 | 0.0 | 0 | 0.0 |
| | 4-6 times a week | 0 | 0.0 | 0 | 0.0 |
| | 2-3 times a week | 2 | 1.9 | 0 | 0.0 |
| | once a week | 5 | 4.8 | 2 | 5.7 |
| | once a two weeks | 23 | 22.1 | 10 | 28.6 |
| | never | 55 | 52.9 | 18 | 51.4 |
| | no data | 19 | 18.3 | 5 | 14.3 |
| trout | | | | | |
| | everyday | 0 | 0.0 | 0 | 0.0 |
| | 4-6 times a week | 0 | 0.0 | 0 | 0.0 |
| | 2-3 times a week | 0 | 0.0 | 0 | 0.0 |
| | once a week | 1 | 1.0 | 0 | 0.0 |
| | once a two weeks | 8 | 7.7 | 5 | 14.3 |
| | never | 76 | 73.1 | 25 | 71.4 |
| | no data | 19 | 18.3 | 5 | 14.3 |
| cod | | | | | |
| | everyday | 0 | 0.0 | 0 | 0.0 |
| | 4-6 times a week | 0 | 0.0 | 0 | 0.0 |
| | 2-3 times a week | 0 | 0.0 | 1 | 2.9 |
| | once a week | 2 | 1.9 | 4 | 11.4 |
| | once a two weeks | 20 | 19.2 | 10 | 28.6 |
| | never | 63 | 60.6 | 15 | 42.9 |
| | no data | 19 | 18.3 | 5 | 14.3 |
| herring | | | | | |
| | everyday | 0 | 0.0 | 0 | 0.0 |
| | 4-6 times a week | 2 | 1.9 | 0 | 0.0 |
| | 2-3 times a week | 2 | 1.9 | 2 | 5.7 |
| | once a week | 4 | 3.8 | 2 | 5.7 |
| | once a two weeks | 17 | 16.3 | 4 | 11.4 |
| | never | 60 | 57.7 | 22 | 62.9 |
| | no data | 19 | 18.3 | 5 | 14.3 |
| sardine | | | | | |
| | everyday | 0 | 0.0 | 0 | 0.0 |
| | 4-6 times a week | 0 | 0.0 | 0 | 0.0 |
| | 2-3 times a week | 0 | 0.0 | 0 | 0.0 |
| | once a week | 0 | 0.0 | 0 | 0.0 |
| | once a two weeks | 1 | 1.0 | 0 | 0.0 |
| | never | 84 | 80.8 | 30 | 85.7 |
| | no data | 19 | 18.3 | 5 | 14.3 |
| eel | | | | | |
| | everyday | 0 | 0.0 | 0 | 0.0 |
| | 4-6 times a week | 0 | 0.0 | 0 | 0.0 |
| | 2-3 times a week | 0 | 0.0 | 0 | 0.0 |

| | | | | | |
|---|---|---|---|---|---|
| | once a week | 0 | 0.0 | 0 | 0.0 |
| | once a two weeks | 0 | 0.0 | 0 | 0.0 |
| | never | 85 | 81.7 | 30 | 85.7 |
| | no data | 19 | 18.3 | 5 | 14.3 |
| halibut | | | | | |
| | everyday | 0 | 0.0 | 0 | 0.0 |
| | 4-6 times a week | 0 | 0.0 | 0 | 0.0 |
| | 2-3 times a week | 0 | 0.0 | 0 | 0.0 |
| | once a week | 0 | 0.0 | 0 | 0.0 |
| | once a two weeks | 1 | 1.0 | 1 | 2.9 |
| | never | 84 | 80.8 | 29 | 82.9 |
| | no data | 19 | 18.3 | 5 | 14.3 |
| sprat | | | | | |
| | everyday | 0 | 0.0 | 0 | 0.0 |
| | 4-6 times a week | 0 | 0.0 | 0 | 0.0 |
| | 2-3 times a week | 0 | 0.0 | 0 | 0.0 |
| | once a week | 2 | 1.9 | 0 | 0.0 |
| | once a two weeks | 5 | 4.8 | 0 | 0.0 |
| | never | 78 | 75.0 | 30 | 85.7 |
| | no data | 19 | 18.3 | 5 | 14.3 |
| pollock | | | | | |
| | everyday | 0 | 0.0 | 0 | 0.0 |
| | 4-6 times a week | 0 | 0.0 | 0 | 0.0 |
| | 2-3 times a week | 0 | 0.0 | 0 | 0.0 |
| | once a week | 1 | 1.0 | 0 | 0.0 |
| | once a two weeks | 0 | 0.0 | 2 | 5.7 |
| | never | 84 | 80.8 | 28 | 80.0 |
| | no data | 19 | 18.3 | 5 | 14.3 |

**Table S4.** Frequency of consumption of nuts in healthy pregnancies and pregnancies complicated by gestational diabetes mellitus (GDM).

| | | **Healthy pregnancies** | | **GDM** | |
|---|---|---|---|---|---|
| | | **n = 104** | | **n = 35** | |
| hazelnuts | | n | % | n | % |
| | everyday | 0 | 0.0 | 1 | 2.9 |
| | 4-6 times a week | 3 | 2.9 | 0 | 0.0 |
| | 2-3 times a week | 4 | 3.8 | 5 | 14.3 |
| | once a week | 4 | 3.8 | 3 | 8.6 |
| | once a two weeks | 17 | 16.3 | 7 | 20.0 |
| | never | 56 | 53.8 | 14 | 40.0 |
| | no data | 20 | 19.2 | 5 | 14.3 |
| walnuts | | | | | |
| | everyday | 2 | 1.9 | 3 | 8.6 |
| | 4-6 times a week | 0 | 0.0 | 0 | 0.0 |
| | 2-3 times a week | 7 | 6.7 | 1 | 2.9 |
| | once a week | 3 | 2.9 | 4 | 11.4 |
| | once a two weeks | 23 | 22.1 | 6 | 17.1 |
| | never | 49 | 47.1 | 16 | 45.7 |

| | | | | | |
|---|---|---|---|---|---|
| | no data | 20 | 19.2 | 5 | 14.3 |
| cashews | | | | | |
| | everyday | 0 | 0.0 | 0 | 0.0 |
| | 4-6 times a week | 0 | 0.0 | 0 | 0.0 |
| | 2-3 times a week | 4 | 3.8 | 2 | 5.7 |
| | once a week | 1 | 1.0 | 1 | 2.9 |
| | once a two weeks | 11 | 10.6 | 2 | 5.7 |
| | never | 68 | 65.4 | 25 | 71.4 |
| | no data | 20 | 19.2 | 5 | 14.3 |
| pistachios | | | | | |
| | everyday | 0 | 0.0 | 0 | 0.0 |
| | 4-6 times a week | 0 | 0.0 | 0 | 0.0 |
| | 2-3 times a week | 2 | 1.9 | 1 | 2.9 |
| | once a week | 3 | 2.9 | 1 | 2.9 |
| | once a two weeks | 8 | 7.7 | 2 | 5.7 |
| | never | 71 | 68.3 | 26 | 74.3 |
| | no data | 20 | 19.2 | 5 | 14.3 |
| almonds | | | | | |
| | everyday | 4 | 3.8 | 2 | 5.7 |
| | 4-6 times a week | 2 | 1.9 | 1 | 2.9 |
| | 2-3 times a week | 4 | 3.8 | 4 | 11.4 |
| | once a week | 3 | 2.9 | 4 | 11.4 |
| | once a two weeks | 16 | 15.4 | 6 | 17.1 |
| | never | 55 | 52.9 | 13 | 37.1 |
| | no data | 20 | 19.2 | 5 | 14.3 |
| peanuts | | | | | |
| | everyday | 1 | 1.0 | 0 | 0.0 |
| | 4-6 times a week | 2 | 1.9 | 0 | 0.0 |
| | 2-3 times a week | 1 | 1.0 | 1 | 2.9 |
| | once a week | 1 | 1.0 | 2 | 5.7 |
| | once a two weeks | 5 | 4.8 | 1 | 2.9 |
| | never | 74 | 71.2 | 26 | 74.3 |
| | no data | 20 | 19.2 | 5 | 14.3 |

**Table S5.** Frequency of consumption of fats and fat-containing products in healthy pregnancies and pregnancies complicated by gestational diabetes mellitus (GDM).

| | | **Healthy pregnancies n = 104** | | **GDM n = 35** | |
|---|---|---|---|---|---|
| margarine | | n | % | n | % |
| | everyday | 17 | 16.3 | 10 | 28.6 |
| | 4-6 times a week | 4 | 3.8 | 1 | 2.9 |
| | 2-3 times a week | 2 | 1.9 | 2 | 5.7 |
| | once a week | 0 | 0.0 | 1 | 2.9 |
| | once a two weeks | 0 | 0.0 | 1 | 2.9 |
| | never | 61 | 58.7 | 15 | 42.9 |
| | no data | 20 | 19.2 | 5 | 14.3 |
| soft margarine | | | | | |
| | everyday | 4 | 3.8 | 2 | 5.7 |

| | | | | | |
|---|---|---|---|---|---|
| | 4-6 times a week | 1 | 1.0 | 0 | 0.0 |
| | 2-3 times a week | 1 | 1.0 | 0 | 0.0 |
| | once a week | 0 | 0.0 | 0 | 0.0 |
| | once a two weeks | 0 | 0.0 | 0 | 0.0 |
| | never | 78 | 75.0 | 28 | 80.0 |
| | no data | 20 | 19.2 | 5 | 14.3 |
| butter | | | | | |
| | everyday | 39 | 37.5 | 9 | 25.7 |
| | 4-6 times a week | 10 | 9.6 | 1 | 2.9 |
| | 2-3 times a week | 9 | 8.7 | 2 | 5.7 |
| | once a week | 0 | 0.0 | 1 | 2.9 |
| | once a two weeks | 1 | 1.0 | 2 | 5.7 |
| | never | 26 | 25.0 | 15 | 42.9 |
| | no data | 19 | 18.3 | 5 | 14.3 |
| butter-margarine mix | | | | | |
| | everyday | 2 | 1.9 | 0 | 0.0 |
| | 4-6 times a week | 0 | 0.0 | 0 | 0.0 |
| | 2-3 times a week | 2 | 1.9 | 0 | 0.0 |
| | once a week | 0 | 0.0 | 0 | 0.0 |
| | once a two weeks | 1 | 1.0 | 0 | 0.0 |
| | never | 79 | 76.0 | 30 | 85.7 |
| | no data | 20 | 19.2 | 5 | 14.3 |
| lard | | | | | |
| | everyday | 0 | 0.0 | 0 | 0.0 |
| | 4-6 times a week | 0 | 0.0 | 0 | 0.0 |
| | 2-3 times a week | 3 | 2.9 | 0 | 0.0 |
| | once a week | 0 | 0.0 | 0 | 0.0 |
| | once a two weeks | 1 | 1.0 | 0 | 0.0 |
| | never | 80 | 76.9 | 30 | 85.7 |
| | no data | 20 | 19.2 | 5 | 14.3 |
| mayonnaise | | | | | |
| | everyday | 1 | 1.0 | 0 | 0.0 |
| | 4-6 times a week | 5 | 4.8 | 1 | 2.9 |
| | 2-3 times a week | 8 | 7.7 | 1 | 2.9 |
| | once a week | 7 | 6.7 | 5 | 14.3 |
| | once a two weeks | 24 | 23.1 | 4 | 11.4 |
| | never | 39 | 37.5 | 19 | 54.3 |
| | no data | 20 | 19.2 | 5 | 14.3 |

**Table S6.** Frequency of consumption of eggs in healthy pregnancies and pregnancies complicated by gestational diabetes mellitus (GDM).

| | | Healthy pregnancies | | GDM | |
|---|---|---|---|---|---|
| | | n = 104 | | n = 35 | |
| eggs | | n | % | n | % |
| | everyday | 7 | 6.7 | 2 | 5.7 |
| | 4-6 times a week | 1 | 0.9 | 1 | 2.9 |
| | 2-3 times a week | 27 | 26.0 | 13 | 37.1 |
| | once a week | 36 | 34.6 | 10 | 28.6 |
| | once a two weeks | 9 | 8.7 | 4 | 11.4 |
| | never | 5 | 4.8 | 0 | 0.0 |
| | no data | 19 | 18.3 | 5 | 14.3 |

**Table S7.** Frequency of consumption of cereals and seeds in healthy pregnancies and pregnancies complicated by gestational diabetes mellitus (GDM).

| | | Healthy pregnancies | | GDM | |
|---|---|---|---|---|---|
| | | n = 104 | | n = 35 | |
| oatmeal | | n | % | n | % |
| | everyday | 5 | 4.8 | 1 | 2.9 |
| | 4-6 times a week | 5 | 4.8 | 0 | 0.0 |
| | 2-3 times a week | 12 | 11.5 | 6 | 17.1 |
| | once a week | 14 | 13.5 | 6 | 17.1 |
| | once a two weeks | 4 | 3.8 | 3 | 8.6 |
| | never | 44 | 42.3 | 14 | 40.0 |
| | no data | 20 | 19.2 | 5 | 14.3 |
| cornflakes | | | | | |
| | everyday | 7 | 6.7 | 1 | 2.9 |
| | 4-6 times a week | 1 | 1.0 | 1 | 2.9 |
| | 2-3 times a week | 10 | 9.6 | 0 | 0.0 |
| | once a week | 3 | 2.9 | 1 | 2.9 |
| | once a two weeks | 3 | 2.9 | 0 | 0.0 |
| | never | 60 | 57.7 | 27 | 77.1 |
| | no data | 20 | 19.2 | 5 | 14.3 |
| rice flakes | | | | | |
| | everyday | 0 | 0.0 | 0 | 0.0 |
| | 4-6 times a week | 0 | 0.0 | 0 | 0.0 |
| | 2-3 times a week | 0 | 0.0 | 0 | 0.0 |
| | once a week | 2 | 1.9 | 0 | 0.0 |
| | once a two weeks | 0 | 0.0 | 0 | 0.0 |
| | never | 82 | 78.8 | 30 | 85.7 |
| | no data | 20 | 19.2 | 5 | 14.3 |
| sunflower seeds | | | | | |
| | everyday | 2 | 1.9 | 1 | 2.9 |
| | 4-6 times a week | 1 | 1.0 | 2 | 5.7 |
| | 2-3 times a week | 3 | 2.9 | 1 | 2.9 |
| | once a week | 4 | 3.8 | 4 | 11.4 |
| | once a two weeks | 10 | 9.6 | 3 | 8.6 |
| | never | 64 | 61.5 | 19 | 54.3 |

| | | | | | |
|---|---|---|---|---|---|
| | no data | 20 | 19.2 | 5 | 14.3 |
| pumpkin seeds | | | | | |
| | everyday | 1 | 1.0 | 1 | 2.9 |
| | 4-6 times a week | 2 | 1.9 | 0 | 0.0 |
| | 2-3 times a week | 0 | 0.0 | 0 | 0.0 |
| | once a week | 3 | 2.9 | 3 | 8.6 |
| | once a two weeks | 7 | 6.7 | 0 | 0.0 |
| | never | 71 | 68.3 | 0 | 0.0 |
| | no data | 20 | 19.2 | 5 | 14.3 |
| wheat bread | | | | | |
| | everyday | 6 | 5.8 | 1 | 2.9 |
| | 4-6 times a week | 1 | 1.0 | 0 | 0.0 |
| | 2-3 times a week | 1 | 1.0 | 0 | 0.0 |
| | once a week | 0 | 0.0 | 0 | 0.0 |
| | once a two weeks | 0 | 0.0 | 0 | 0.0 |
| | never | 75 | 72.1 | 29 | 82.9 |
| | no data | 21 | 20.2 | 5 | 14.3 |
| rye bread | | | | | |
| | everyday | 22 | 21.2 | 13 | 37.1 |
| | 4-6 times a week | 7 | 6.7 | 2 | 5.7 |
| | 2-3 times a week | 2 | 1.9 | 1 | 2.9 |
| | once a week | 2 | 1.9 | 0 | 0.0 |
| | once a two weeks | 0 | 0.0 | 1 | 2.9 |
| | never | 51 | 49.0 | 13 | 37.1 |
| | no data | 20 | 19.2 | 5 | 14.3 |
| whole wheat bread | | | | | |
| | everyday | 18 | 17.3 | 8 | 22.9 |
| | 4-6 times a week | 12 | 11.5 | 2 | 5.7 |
| | 2-3 times a week | 4 | 3.8 | 2 | 5.7 |
| | once a week | 7 | 6.7 | 1 | 2.9 |
| | once a two weeks | 4 | 3.8 | 0 | 0.0 |
| | never | 39 | 37.5 | 17 | 48.6 |
| | no data | 20 | 19.2 | 5 | 14.3 |

**Table S8.** Frequency of consumption of selected food products not included in previous categories in healthy pregnancies and pregnancies complicated by gestational diabetes mellitus (GDM).

| | | **Healthy pregnancies** | | **GDM** | |
|---|---|---|---|---|---|
| | | **n = 104** | | **n = 35** | |
| avocado | | n | % | n | % |
| | everyday | 1 | 1.0 | 0 | 0.0 |
| | 4-6 times a week | 0 | 0.0 | 0 | 0.0 |
| | 2-3 times a week | 1 | 1.0 | 1 | 2.9 |
| | once a week | 3 | 2.9 | 1 | 2.9 |
| | once a two weeks | 4 | 3.8 | 3 | 8.6 |
| | never | 75 | 72.1 | 25 | 71.4 |
| | no data | 20 | 19.2 | 5 | 14.3 |
| lettuce | | | | | |
| | everyday | 8 | 7.7 | 6 | 17.1 |

| | | | | | |
|---|---|---|---|---|---|
| | 4-6 times a week | 2 | 1.9 | 0 | 0.0 |
| | 2-3 times a week | 17 | 16.3 | 6 | 17.1 |
| | once a week | 14 | 13.5 | 7 | 20.0 |
| | once a two weeks | 13 | 12.5 | 1 | 2.9 |
| | never | 30 | 28.8 | 10 | 28.6 |
| | no data | 20 | 19.2 | 5 | 14.3 |
| spinach | | | | | |
| | everyday | 0 | 0.0 | 0 | 0.0 |
| | 4-6 times a week | 0 | 0.0 | 1 | 2.9 |
| | 2-3 times a week | 4 | 3.8 | 0 | 0.0 |
| | once a week | 10 | 9.6 | 7 | 20.0 |
| | once a two weeks | 13 | 12.5 | 6 | 17.1 |
| | never | 57 | 54.8 | 16 | 45.7 |
| | no data | 20 | 19.2 | 5 | 14.3 |
| tofu | | | | | |
| | everyday | 0 | 0.0 | 0 | 0.0 |
| | 4-6 times a week | 0 | 0.0 | 0 | 0.0 |
| | 2-3 times a week | 0 | 0.0 | 0 | 0.0 |
| | once a week | 0 | 0.0 | 0 | 0.0 |
| | once a two weeks | 0 | 0.0 | 1 | 2.9 |
| | never | 84 | 80.8 | 29 | 82.9 |
| | no data | 20 | 19.2 | 5 | 14.3 |
| vitamin supplementation | | | | | |
| | yes | 66 | 63.5 | 21 | 60.0 |
| | no | 34 | 32.7 | 14 | 40.0 |
| | no data | 4 | 3.8 | 0 | 0.0 |
| potato fries | | | | | |
| | yes | 37 | 35.6 | 8 | 22.9 |
| | no | 53 | 51.0 | 24 | 68.6 |
| | no data | 14 | 13.5 | 3 | 8.6 |
| chips | | | | | |
| | yes | 24 | 23.1 | 4 | 11.4 |
| | no | 66 | 63.5 | 28 | 80.0 |
| | no data | 14 | 13.5 | 3 | 8.6 |

**Table S9.** PCA loadings for fatty acid composition of maternal serum lipids.

| Variable | PC1 loading | PC2 loading |
|---|---|---|
| n6 PUFA | 0.334 | 0.147 |
| LA | 0.325 | 0.087 |
| C16:1 | -0.322 | -0.101 |
| C14:0 | -0.303 | 0.054 |
| PUFA | 0.3 | 0.233 |
| C17:1 | -0.278 | -0.074 |
| n6/n3 PUFA | 0.26 | -0.241 |
| SFA | -0.25 | -0.052 |
| C14:1 | -0.233 | 0.064 |
| C16:0 | -0.231 | -0.137 |
| t9,t12 C18:2 | -0.193 | 0.076 |

| | | |
|---|---|---|
| C15:0 | -0.182 | 0.161 |
| GLA | -0.146 | 0.137 |
| C20:1 | -0.109 | 0.151 |
| DHA | -0.107 | 0.366 |
| n3 PUFA | -0.105 | 0.383 |
| C20:2 | 0.098 | 0.074 |
| MUFA | -0.093 | -0.289 |
| AA | 0.092 | 0.205 |
| C12:0 | -0.092 | 0.079 |
| EPA | -0.073 | 0.345 |
| C17:0 | -0.068 | 0.179 |
| C22:0 | -0.058 | 0.112 |
| C23:0 | 0.056 | 0.168 |
| C22:1 | -0.054 | 0.038 |
| C24:0 | -0.034 | 0.016 |
| C20:0 | -0.033 | -0.06 |
| ALA | -0.033 | 0.062 |
| Oleic acid (C18:1n9) | 0.002 | -0.267 |
| C18:0 | 0.002 | 0.226 |

PC1 explained 19.8% of variance and PC2 explained 15.0%.
